# Torted and Ruptured Wandering Spleen Presenting as a Surgical Emergency in Pregnancy

**DOI:** 10.1100/tsw.2004.208

**Published:** 2004-12-06

**Authors:** A. Parvaiz, S. Chandran, A. Karim, K. Kumar, P. Jeffrey, N.R.F. Lagattolla

**Affiliations:** Department of Surgery, Dorset County Hospital, Williams Avenue, Dorchester DT1 2JY, UK

**Keywords:** wandering spleen, splenic infarction, hypermobile spleen

## Abstract

Wandering spleen (WS) is characterised by incomplete fixation of the spleen to its supporting linorenal and gastrosplenic ligaments. It can predispose to life-threatening complications due to torsion of its vascular pedicle, splenic infarction, portal hypertension, and haemorrhage. A 27-year-old, 36-week prima gravida underwent emergency caesarean section for tachycardia and hypotension. A healthy baby girl was delivered. However, she remained shocked despite aggressive fluid therapy and intraoperatively it was noted that there was significant intraperitoneal bleeding and the on-call surgical team was summoned. Midline laparotomy revealed a lacerated, infarcted, hypermobile spleen found with free intraperitoneal bleeding. The unsalvageable spleen was resected and the patient went on to make an excellent recovery. The aetiology of WS is contentious. With an increased frequency among multiparous females of reproductive age, some suggest the hormonal effects of pregnancy as contributing factors. Clinical presentations range from an asymptomatic abdominal mass to acute abdominal pain with hypovolaemic shock. WS poses a serious threat to life due to thrombosis, bleeding, or infarction. Ultrasound scan and CT scan are equally effective in the diagnosis. Patients with asymptomatic WS should be treated with elective splenopexy, however, in the acute presentation, splenectomy is the procedure of choice.

## INTRODUCTION

Wandering spleen (WS) is a rare entity characterised by the incomplete fixation of the spleen by its supporting linorenal and gastrosplenic ligaments. It is most commonly found in females of reproductive age, presenting as an asymptomatic abdominal mass or more commonly with acute, chronic, or intermittent abdominal pain[1]. WS can predispose to life-threatening complications due to torsion of its vascular pedicle, splenic infarction, portal hypertension, and haemorrhage.

We report a case of a WS in a 36-week pregnant female who presented as an emergency, following intraperitoneal haemorrhage at caesarean section, requiring emergency splenectomy. We believe that this presentation of WS with intraperitoneal bleeding in a near-term pregnant female has not been described before.

## CASE REPORT

A 27-year-old, prima gravida at 36-weeks gestation presented to her obstetrician with backache, right shoulder tip pain, dizziness, and syncope. She had attended the antenatal clinic at 32 weeks complaining of intermittent abdominal pain and vaginal bleeding. An ultrasound scan at this point confirmed an anteriorly lying placenta with no foetal abnormalities. Her full blood count demonstrated mild thrombocytopenia with a platelet count of 109. She was reassured and treated symptomatically with analgesia.

On emergency admission, she was pale with cold extremities. She was tachycardic at 130 beats per min with a blood pressure of 90/40 mmHg. Her CTG showed prolonged periods of foetal bradycardia, suggestive of placental abruption and she resultantly underwent emergency caesarean section. There were no intraoperative complications during the procedure and a healthy baby girl was delivered. However, the patient remained hypotensive and tachycardic despite aggressive fluid therapy and it was retrospectively noted that there was significantly more intraperitoneal bleeding than could have be accounted for by the caesarean section alone. The on-call surgical team was therefore summoned for advice.

Midline laparotomy was performed and a lacerated, partially infarcted, hypermobile spleen was found with free intraperitoneal bleeding (Figs. 1 and 2). The spleen was unsalvageable and emergency splenectomy was performed. The patient made an excellent recovery postoperatively and was discharged home at 6 days.

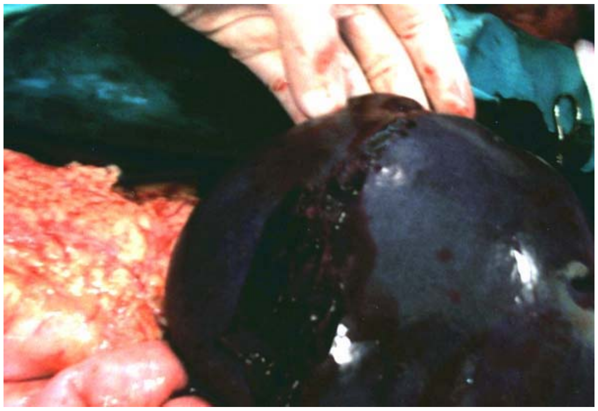

**FIGURE 1**. Lacerated, infarcted, hypermobile spleen.

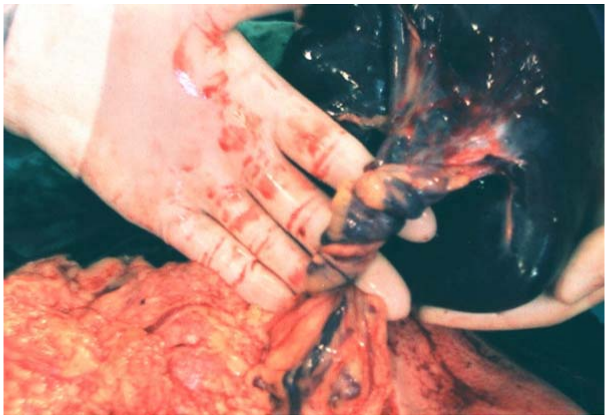

**FIGURE 2**. Torted vascular pedicle of wandering spleen.

## DISCUSSION

The aetiology of WS is contentious. Considering the increased incidence of this rare condition among multiparous females of reproductive age, some suggest abdominal wall laxity and the hormonal effects of pregnancy as contributing factors in the development of this rare condition[2]. Splenomegly has been implicated as a possible cause, but there is no higher incidence of WS in areas where splenomegaly is endemic[2,3]. Various studies have suggested that the dorsal mesogastrium fails to develop properly resulting in abnormalities of the spleens supporting linorenal ligament, permitting splenic hypermobility[5,6,7]. The clinical presentation ranges from an asymptomatic abdominal mass to more commonly acute intermittent or chronic abdominal pain. There are reports of this condition presenting as reversible hypersplenism[8] and acute pancreatitis[9]. In the acute presentation, WS poses a serious threat to life due to thrombosis, bleeding, or infarction. Ultrasound scan and CT scan are equally effective in the diagnosis of this condition. Patients with a WS causing an asymptomatic abdominal or pelvic mass should be treated with splenopexy. However, if the presentation is acute, splenectomy is the procedure of choice either with a conventional open or laparoscopic approach[10].
